# Microglia receptors and their implications in the response to amyloid β for Alzheimer’s disease pathogenesis

**DOI:** 10.1186/1742-2094-11-48

**Published:** 2014-03-13

**Authors:** Deborah Doens, Patricia L Fernández

**Affiliations:** 1 Centro de Biología Molecular y Celular de Enfermedades, Instituto de Investigaciones Científicas y Servicios de Alta Tecnología (INDICASAT-AIP), Edificio 219, Clayton, Ciudad del Saber, República de Panamá; 2 Department of Biotechnology, Acharya Nagarjuna University, Guntur, India

**Keywords:** Cytokines, Inflammation, Microglia, Receptor

## Abstract

Alzheimer’s disease (AD) is a major public health problem with substantial economic and social impacts around the world. The hallmarks of AD pathogenesis include deposition of amyloid β (Aβ), neurofibrillary tangles, and neuroinflammation. For many years, research has been focused on Aβ accumulation in senile plaques, as these aggregations were perceived as the main cause of the neurodegeneration found in AD. However, increasing evidence suggests that inflammation also plays a critical role in the pathogenesis of AD. Microglia cells are the resident macrophages of the brain and act as the first line of defense in the central nervous system. In AD, microglia play a dual role in disease progression, being essential for clearing Aβ deposits and releasing cytotoxic mediators. Aβ activates microglia through a variety of innate immune receptors expressed on these cells. The mechanisms through which amyloid deposits provoke an inflammatory response are not fully understood, but it is believed that these receptors cooperate in the recognition, internalization, and clearance of Aβ and in cell activation. In this review, we discuss the role of several receptors expressed on microglia in Aβ recognition, uptake, and signaling, and their implications for AD pathogenesis.

## Background

Alzheimer’s disease (AD) is a neurodegenerative disorder characterized by a progressive decline in cognitive and functional abilities. According to the World Health Organization, more than 35 million people have dementia and this number is expected to increase in the coming years [1]. The neuropathological hallmarks of AD include extracellular Aβ deposits, intracellular neurofibrillary tangles, and marked inflammation [2-4]. Aβ deposition and tau protein are found in different areas of the brain, leading to synaptic dysfunction, mitochondrial damage, activation of microglia, and neuronal death [5,6]. Inflammation in AD is characterized by reactive microglia surrounding Aβ plaques, which maintain an inflammatory status by secreting proinflammatory mediators, contributing to neuronal loss.

Microglia constitute the lesser portion of the total glial cell population within the brain and are found in a resting state in the healthy central nervous system (CNS) [7]. Under pathological conditions, activated microglia undergo morphological changes and produce cytokines and chemokines that affect surrounding cells [8]. In AD, microglia cells play an important role in disease progression by clearing Aβ deposits, initiating phagocytic activity, and releasing cytotoxic mediators. Microglia activated by Aβ *in vitro* induce the expression of proinflammatory cytokines including interleukin (IL)-1β, IL-6, IL-8, tumor necrosis factor-α (TNF-α), chemokines and reactive oxygen and nitrogen species, all of which cause neuronal damage [9-11].

**Figure 1:**
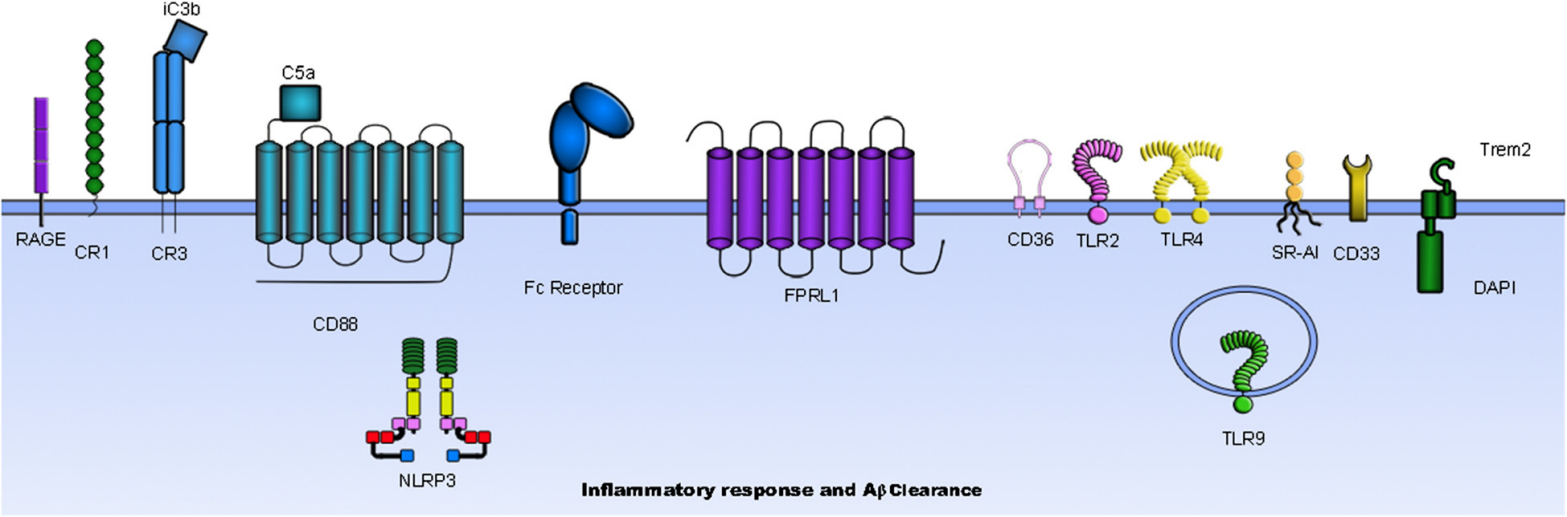
**Microglia receptors involved in the amyloid cascade.** A variety of microglia receptors are involved in Aβ clearance and in triggering an inflammatory response. Some receptors (RAGE, NLRP3) are mainly implicated in the generation of an inflammatory response by triggering a signaling cascade that results in the production of proinflammatory mediators. Other receptors (SR-AI, TREM2) are involved in the clearance of Aβ by inducing internalization of Aβ fibrils. Some receptors (complement receptors, Fc receptors, FPRL1/FPR2, CD36, TLRs) are involved in both processes. CD33 seems to promote Aβ accumulation.

The mechanisms through which amyloid deposits provoke inflammation are not fully understood. Microglia cells express several receptors that cooperate in the recognition, internalization, and clearance of Aβ and in cell activation. Microglia receptors, such as scavenger receptors (SR-AI/II), CD36, RAGE (receptor for advanced glycosylation endproducts), Fc receptors, TLRs (toll-like receptors), and complement receptors are involved in these processes [12-14] (Figure 1). This review will examine the various roles of microglia receptors in the amyloid cascade, and the implications for AD.

## Complement receptors

The complement system is formed of a number of soluble and membrane-associated proteins that interact to opsonize microorganisms and to induce an inflammatory response that contributes to the resolution of the infectious process [15]. The association of the complement system with AD pathology has been known since the 1980s [16]. Proteins of the complement system have been associated with senile plaques in the brains of AD individuals [17]. Several proteins of the complement system and their corresponding mRNAs are upregulated in the brains of AD patients and seem to be involved in Aβ induced inflammation, senile plaque formation, and Aβ phagocytosis [18].

The activation of the complement system takes place via three main pathways known as classical, alternative, and MB-lectin [18]. Fibrillar Aβ (fAβ) activates the classical as well as the alternative pathways with consequent C3 activation, C5a production, and membrane attack complex (MAC) formation [19]. The role of the complement system in the removal of the infectious agent occurs through the activation of a variety of receptors including CR1 (CD35), CR2 (CD21), CR3 (CD11b/CD18), CR4 (CD11c/CD18), and C5aR (CD88 and C5L2). Some of these receptors play a prominent role in the inflammatory response induced in AD [12].

CR1 is a transmembrane receptor that plays a major role in the regulation of the complement cascade activation. CR1 binds the complement factors C3b and C4b; high levels of this receptor have been detected in the cerebrospinal fluid (CSF) of AD patients [20]. A recent genome-wide association study in a Caucasian population showed an association of some variants of CR1 with late-onset AD risk, which has drawn increased attention to the role of this receptor in the pathogenesis of AD [21]. Those CR1 variants were further correlated with characteristic neuroimaging markers of the disease [22]. The association between CR1 and AD risk has been reproduced in case-control studies in other populations [23,24].

Activated microglia have increased expression levels of CR1; activation of this receptor induces neuronal death [25]. These detrimental effects appear to be associated with enhanced superoxide generation and TNF-α and IL-1β production. CR1 expressed on erythrocytes participates in the clearance of peripheral Aβ, suggesting that CR1 may play a role in the removal of Aβ in AD [26]. Polymorphisms in the *CR1* locus, which constitute a risk for AD, have been correlated with increased levels of Aβ in the CSF [27]. Owing to the role of CR1 in the clearance of Aβ and regulation of complement activation, it has been suggested that this receptor may have a beneficial effect on the pathogenesis of AD [28], although the mechanisms are unknown.

The complement factor C3 is an essential component of the complement system. It induces phagocytosis of pathogens through interactions with the CR3 receptor. CR3, also known as Mac-1, is expressed in microglia, and upregulation of this receptor has been detected in the brains of AD individuals [29]. Studies have shown that CR3 appears to be involved in the uptake and clearance of Aβ *in vivo* and *in vitro*[30-32]. Fu *et al.* have recently suggested that CR3 acts together with the scavenger receptor A (SR-A) in the uptake of Aβ [32]. They also showed that murine microglia treated with ligands of SR-A reduced their capacity for Aβ uptake.

CR3 has also been shown to colocalize with Aβ plaques in the brains of AD patients, providing evidence for a possible direct CR3-Aβ interaction [29]. CR3 is partially involved in Aβ activation of microglia *in vivo* and *in vitro* and is implicated in microglia free-radical generation in response to Aβ [33]. These effects appear to be dependent on the binding of Aβ to CR3. Reduced activation was observed in microglia obtained from knockout mice for CR3 (MAC1^-/-^) after *in vitro* Aβ challenge compared with microglia derived from control mice [33]. The use of CR3 antagonists has been proposed as a potential therapeutic approach for AD treatment aimed at reducing the activation of proinflammatory mediators and reactive oxygen species in microglia exposed to Aβ [33].

C5a is a highly proinflammatory molecule generated in the process of complement activation. CD88 is a receptor for C5a expressed on the surface of innate immune cells, including microglia. The interaction between C5a and CD88 leads to the production of inflammatory cytokines, reactive oxygen species, and bioactive amines, among other inflammatory mediators [34]. CD88 is a chemotactic receptor and is involved in the *in vitro* and *in vivo* recruitment and activation of microglia [34]. Increased levels of CD88 have been detected in microglia located in the vicinity of amyloid plaques in the brains of AD mouse models [35]. The co-stimulation of human monocytes with Aβ and C5a induces an increase in IL-1β and IL-6 secretion [36], potentially through a mechanism involving cooperation between microglia receptors. The detrimental role of CD88 in AD has been demonstrated by the use of an antagonist of this receptor, which decreased Aβ plaques, diminished glia activation, and improved contextual memory in two transgenic AD mouse models [37]. A second receptor for C5a, C5L2, has recently been described as having an increased expression in AD brains compared with normal-aged individuals [38], although its role in AD pathology is still unknown.

Despite this evidence, suggesting a detrimental role of the complement system in AD, some studies have shown that it has beneficial effects in the course of the disease. For example, APP mice deficient in the complement component C3 exhibited increased Aβ deposition in the brain, associated with a prominent neuronal loss at 17 months of age [30]. Similarly, overexpression of an inhibitor of the complement in a transgenic mouse model of AD triggered higher deposition of Aβ and increased neurodegeneration compared with controls [39]. Increased C3 mRNA levels have been associated with a reduction in Aβ deposition in mice expressing the human amyloid precursor protein (hAPP) and TGF-β [39]. Neuroprotective functions have also been attributed to the products of the complements C3a and C5a [40]. Overall, these results suggest that activation of complement receptors may promote the clearance of Aβ, potentially reducing Aβ accumulation and neurodegeneration in AD. More studies are needed to clarify the role of the complement system in the brain and test its potential application to the design of novel AD treatments.

## Fc receptors

Fc receptors (FcRs) bind the constant domain (Fc) of immunoglobulins (Ig). Specific FcRs exist for each isotype class and sub-class of Ig; for example, IgA is the ligand for FcαR, IgD for FcδR, IgM for FcμR, IgE for FcϵR, and IgG for FcγR [41]. FcR engagement in immune cells activates phagocytosis, degranulation and cytokine and chemokine secretion. FcRs are expressed in brain cells, including microglia, which express all classes of FcRs [41]. Mitogen-activated protein kinases (MAPKs), nuclear factor-κB, Src, and Syk kinases are all involved in the activation of FcγR in microglia [42,43]. The role of FcRs expressed in microglia in AD and healthy brains was first suggested by Peress *et al.*[44]. There is evidence that Ig bound to neuronal antigens activates a microglial inflammatory response through FcRs expressed on these cells, which may be responsible for the neurodegeneration observed in AD [45].

Active and passive Aβ immunization in studies with AD animal models has demonstrated an effect of anti-Aβ antibodies on Aβ clearance and the reduction of cognitive decline [46-48]. FcRs in microglia have been shown to mediate Aβ phagocytosis in the presence of antibodies [47,48]. In contrast, other studies have shown that increased Aβ clearance *in vivo* in the presence of anti-Aβ antibodies is not dependent on FcR-mediated phagocytosis [49]. In addition to Fc-mediated phagocytosis, a non-Fc-mediated disruption of plaque structure occurs *in vivo* in the presence of antibodies bound to Aβ deposits [50]. Both Aβ clearance pathways involving antibodies do not seem to be mutually exclusive and might occur in parallel or sequentially.

In addition, increased levels of IgG in the CSF of patients with AD have been reported [51,52]. In pathological conditions in which the integrity of the blood brain barrier has been compromised, as in the case for AD, Igs may pass through the blood brain barrier, and thereby mediate neurotoxicity and inflammation [53]. Some authors have proposed that there is intra-blood-brain-barrier synthesis of Igs in patients with AD [51]. However, the role of FcRs in the activation of microglia by naturally produced antibodies during the course of AD is not well understood.

FcγRs expressed on neurons have also been implicated in neurotoxicity and inflammation occurring directly in these cells [54,55]. Likewise, a recent study demonstrated that there is a physical interaction between FcγRIIb and Aβ_42_, which mediates neurotoxicity [56]. Together, these results do not rule out the possibility of a potential ‘crosstalk’ between FcγRs and other Aβ receptors in the cell.

Several hypotheses have tried to explain the use of Aβ immunotherapy as a treatment strategy for AD [57]. One of these hypotheses concerns the role of FcR in mediating phagocytosis of opsonized Aβ by microglia. Several active and passive Aβ immunotherapies are currently being trialed in preclinical and clinical studies. Although these approaches have had an effect on the clearance of Aβ plaques in AD patients, little or no improvement has been observed in cognitive performance once extensive neuronal damage has occurred. This topic has recently been reviewed elsewhere [57-59].

The results presented so far demonstrate the complexity of the role that FcRs play in AD progression. Experimental variability, manifested in the variety of animal models used, the timing of the development of AD-like pathology, the use of different antibodies, doses, and routes of inoculation, and other factors, make it difficult to clarify the capacity of these receptors to modulate the development of the disease. Brain cells’ responses to antibodies, whether or not they are mediated by FcRs, can have multiple effects on CNS function [60]. Further studies are required to understand the role of FcR-mediated Aβ clearance in response to naturally generated antibodies in AD pathogenesis.

## Formyl peptide receptors (FPRs)

The FPRs are receptors for the bacterial chemotactic peptide fMLP [61]. The FPRs are members of a family of seven transmembrane domains, G-protein-coupled receptors, and are involved in host defense against pathogens and endogenous molecules. Two FPRs have been identified in human beings, FPR1 and FPRL1, along with their counterparts FPR1 and FPR2 in mice. FPRL1 interacts with several host-derived chemotactic agonists, including HIV-1 envelope protein, serum amyloid A, and Aβ_42_[61-63].

FPRL1 interacts with Aβ_42_ through the N-terminus as well as a segment between the fourth transmembrane domain and the third extracellular loop [64]. On mononuclear phagocytes, FPRL1 and FPR2 have been identified as functional receptors for Aβ_42_-induced IL-1β and superoxide secretion [61,65]. Aβ induces cell migration and calcium mobilization in HEK293 cells transfected with FPRL1 [61]. The complex Aβ_42_-FPRL1 is internalized into the cytoplasmic compartment of macrophages and HEK293 cells overexpressing FPRL1 [66]. Subtraction of Aβ_42_ from the culture results in a progressive recycling of FPRL1 to the cell membrane, whereas continuous exposure to Aβ_42_ results in intracellular accumulation of Aβ_42_/FPRL1 complex [66]. Further studies have supported the role of FPRL1 and FPR2 in the endocytosis of Aβ_42_[67,68].

The expression of FPR2 is increased in primary microglia and N9 cells after lipopolysaccharide (LPS) treatment [69]. LPS-stimulated microglia cells exhibit calcium mobilization and chemotaxis in response to FPR2 agonists including Aβ_42_. Moreover, the stimulation of microglia cells with IFN-γ increases FPR2 expression levels and cell migration in response to several FPR2 agonist peptides, such as Aβ_42_[70]. These results suggest that endogenous or exogenous agents modulate the response to Aβ by regulating the expression of FPR, and point to potential effects on AD pathology.

FPR2/FPRL1 has been proposed as a potential therapeutic target for AD based on observations that FPR2 antagonists reduced the proinflammatory response induced by Aβ in monocytes [71]. However, most studies demonstrating a role of FPRs in Aβ uptake and microglia activation have been performed *in vitro*; thus, the *in vivo* relevance of this receptor remains uncertain.

## Scavenger receptors

Scavenger receptors (SR) are structurally diverse cell surface receptors that participate in cellular adhesion and uptake of ligands [72]. Goldstein first described these receptors in 1979 as macrophage receptors with the ability to bind and internalize acetylated low-density lipoproteins (acLDL) and a variety of lipids [73,74]. The SR family can be classified into at least eight classes in mammalian species; most of them are related to atherosclerosis pathogenesis [75,76]. Two classes of SR have been described in the CNS. Class A (SR-A) receptors are expressed on microglial and astrocytes and class B scavenger receptor type 2 (also known as CD36) is expressed on microglia and endothelial cells [77,78]. Class A and B SRs have been associated with AD pathogenesis because both are able to bind and internalize Aβ, triggering an inflammatory response [79].

### Scavenger receptor A

SR-A type I is a trimeric receptor with a short cytoplasmic tail, a transmembrane region, an α helical coiled domain, a collagenous-like region and a cysteine-rich domain in the C-terminal position [80]. Three isoforms of SR-A have been identified: SR-AI, SR-AII, and SR-AIII, all of which are generated by alternative splicing of a single gene [81,82]. SR-AI was first described as an acetylated low-density lipoprotein (LDL) receptor but it is now known that it binds to a broad diversity of ligands, such as microbial ligands, acLDL, endotoxins, and Aβ [83-85]. The uptake of ligands by SR-AI is associated with several conditions, including AD and atherosclerosis [85,86].

SR-AI has been detected on activated microglia in the vicinity of senile plaques from human brain tissue [80]. Evidence shows that SR-AI-binding to Aβ promotes Aβ internalization and clearance [85,87,88]. The role of SR-AI in Aβ clearance was demonstrated by reduced Aβ internalization levels found in mouse microglia treated with neutralizing anti-SR-AI antibodies [88]. Moreover, SR-AI expression levels and Aβ clearance are reduced when microglia activation is sustained for a long period of time [85]. In addition, PS1/APP transgenic mice with a SR-AI deficiency have increased Aβ deposition levels in the brain, which are, in turn, associated with an increase in mortality [89]. Thus, owing to its role in Aβ internalization and clearance, the upregulation of SR-AI expression has been proposed as a possible therapeutic target for AD.

### CD36 receptor

CD36 is a type B scavenger receptor found in a variety of cell types, such as macrophages [90], dendritic cells [91], microglia [77], adipocytes [92], platelets [93], endothelial cells [94], and sensory cells of the retina [95]. CD36 was first described as a thrombospondin receptor and as a receptor for other molecules containing the thrombospondin-type repeat domain [96]. The CD36 receptor consists of an extracellular domain and two cytoplasmic fragments containing the C-terminal and N-terminal domains [97]. CD36, considered a pattern recognition receptor, recognizes exogenous molecules, such as microbial components [98], as well as endogenous molecules, such as low-density lipoproteins, oxidized phospholipids [99], apoptotic cells, and Aβ [90]. CD36 has been implicated in the pathogenesis of several diseases, including AD [13], atherosclerosis [100], and malaria [101], and has been identified as an endogenous negative regulator for angiogenesis [94].

The role of CD36 in AD has been demonstrated by its effect on microglia recruitment [102] and activation in response to fAβ [77,102,103]. Decreases in cytokine and chemokine expression (MCP-1, IL-1β, MIP-1α, MIP1β, MIP-2, TNFα, and KC) have been observed in macrophages and microglia from CD36-deficient mice stimulated with fAβ [102]. Notably, there was elevated expression of CD36 in human brains with Aβ deposits, whereas CD36 was undetectable in healthy brains without Aβ deposition [104].

In addition, CD36 forms complexes with other pattern recognition receptors to bind fibrillar proteins. The first complex identified in microglia for fAβ recognition was composed of CD36, α_6_β_1_ integrin and CD47 [105]. Arrangement of this complex was shown to activate a tyrosine-kinase signaling cascade that led to reactive oxygen species (ROS) production, cytokine expression, and phagocytosis induction. Recent evidence indicates that CD36 also forms a complex with TLR4 and TLR6 [106]. CD36 acts as a co-receptor of TLR4 and TLR6, providing signals for assembly of the CD36-TLR4-TLR6 complex and subsequent activation of the TLRs signaling cascades.

In summary, these results demonstrate that CD36 is a key element for fAβ-induced microglia and macrophage activation. Recently, a cell-based assay was developed to screen for small molecules that inhibit binding between Aβ and CD36 [107]. This bioassay identified ursolic acid as an inhibitor of the Aβ-CD36 interaction and ROS production in Chinese hamster ovary cells expressing human CD36. Thus, the inhibition of Aβ-CD36 binding is a potential strategy for interrupting the pathogenic processes induced by Aβ.

## Receptor for advanced glycosylation endproducts (RAGE)

RAGE was originally described as a receptor of advanced glycosylation endproducts (AGE), which is formed when a reduced sugar, such as glucose, reacts with proteins [108]. RAGE was later described as a multiligand receptor member of the immunoglobulin super family, which is able to bind S100 proteins, high mobility group box 1, Aβ peptide, and β-sheet fibrils, among other ligands [109-111]. RAGE is expressed in endothelial cells, macrophages, smooth muscle cells, and neurons [112]. RAGE is implicated in the transport of Aβ through the blood brain barrier [113]. Aβ induces NF-κB activation in neurons, microglia, and endothelial cells, and promotes the production of proinflammatory molecules through the interaction with RAGE [114,115]. Several studies have revealed that neuronal dysfunction and inflammatory processes found in AD are linked to microglia activation by Aβ recognition through RAGE [113,116-118]. Moreover, it has been suggested that RAGE interacts physically and functionally with FPRL1 to transduce signaling in glia cells [119].

Evidence indicates that the interaction between RAGE expressed on brain endothelial cells and Aβ leads to the activation of MAPKs, c-Jun N-terminal kinases, and extracellular signal-regulated kinases (ERKs) [120]. The activation of these pathways promotes endothelial matrix metalloproteinase-2 production, which is associated with the vascular inflammatory responses also found in AD [120]. Evidence suggests that microglia activation by the RAGE-Aβ interaction also involves the p38 MAPK signaling pathways [111,117]. Fang and colleagues demonstrated that microglia overexpression of RAGE in a transgenic AD animal model (transgenic mAPP) increased the production of proinflammatory mediators such as IL-1β and TNF-α after Aβ stimulation. This increase was associated with higher levels of phosphorylated p38 and ERK1/2 [111]. Accordingly, the elevated levels of proinflammatory molecules due to microglia RAGE-Aβ interaction are likely to cause the neuronal damage that leads to deficits in learning and memory. However, early studies demonstrated that RAGE-Aβ interactions on the surface of neurons mediate neurotoxicity by inducing oxidative stress [121].

Some research groups have focused on identifying small molecules that might be able to block the Aβ-RAGE interaction as a possible therapeutic strategy. Pfizer reached phase II clinical trials for the small molecule RAGE-Aβ inhibitor, called PF-04494700, as an AD pharmacotherapeutic [122]. Later, trials were discontinued when it was confirmed that the treatment did not produce significant effects on secondary outcomes. More recently, a small molecule (FPS ZM1) was discovered that was capable of blocking this interaction *in vitro* by binding to the RAGE V domain, inhibiting its ability to recognize Aβ and resulting in a reduction in cellular oxidative stress [123].

## Toll-like receptors (TLRs)

TLRs are a family of membrane proteins that recognize a variety of molecules referred to as danger- and pathogen-associated molecular patterns. Toll receptors were first described in *Drosophila melanogaster* for their role in embryo development and the response to fungal infection in adult flies [124,125]. In mammals, 12 TLRs have been described and are expressed in a variety of cells, including microglia and astrocytes [126,127]. The activation of TLRs triggers different signaling pathways, leading to the production of proinflammatory mediators, such as cytokines, nitric oxide, and ROS [128].

Microglia expression of TLRs in the CNS is crucial as a first line of defense against exogenous and endogenous molecules [126]. Microglia express TLRs 1 to 9, and most of these receptors have been associated with microglia activation and neurotoxicity in both mice and human beings [127,129]. High levels of mRNA for TLR2, TLR4, TLR5, TLR7, and TLR9 have been detected in plaque-associated brain tissue of APP23 transgenic mice [130]. TLRs have been implicated in Aβ signaling, where they trigger an intracellular cascade, resulting in the production of proinflammatory molecules and the uptake and clearance of Aβ [131,132].

TLR4 has traditionally been described as a LPS receptor [133] but is capable of recognizing other endogenous and exogenous molecules [134,135]. Several studies have pointed to the importance of microglia activation through the TLR4 pathway [132,136,137]. In addition to the role of TLR4 in recognizing LPS by microglia, studies have shown its relevance in response to microglia-Aβ activation [138]. The activation of murine microglia by Aβ depends on a functional TLR4 coupled with CD14 and myeloid differentiation protein 2 [138]. This microglia activation was implicated in neurotoxicity based on observations of a decrease in the death of hippocampal neurons cell cultures after contact with supernatant of Aβ-stimulated microglia from TLR4 mutated mice [138].

Microglia cells stimulated with TLR4 ligands, such as LPS, showed an increase in Aβ uptake *in vitro*[132]. In addition, mice with a deficient lipopolysaccharide response (Tlr4^Lps-d^) showed an increase in Aβ load *in vivo* and a decrease in Aβ uptake by microglia *in vitro*[132]. Taken together, these findings suggest that TLR4 might be involved in the clearance of Aβ. Furthermore, *in vivo* experiments with a TLR4-mutated AD mouse model showed spatial learning deficits and elevated levels of Aβ_42_ in the brain [137].

A recent study suggested that the monophosphoryl lipid A (MPL), a TLR4 agonist with lower toxicity than LPS, acts as an Aβ clearance booster [139]. MPL induced a mild inflammatory response in microglia while increasing the ability of these cells to internalize Aβ, a mechanism that involves the activation of p38 and the expression of the SR-AI [139].

Overall, these results suggest different roles for TLR4 signaling, which appear to be associated with both beneficial (clearance of Aβ) and detrimental (neurotoxicity) processes. Different therapeutic approaches for AD can be addressed to overcome the detrimental functions of TLR4. Blocking TLR4 signaling would inhibit microglia activation, thus reducing cytokine production, but would impair Aβ uptake and increase Aβ deposition. On the other hand, the induction of TLR4 signaling through MPL-like activation could increase Aβ uptake with reduced production of proinflammatory cytokines.

TLR2 has also been implicated in the inflammatory response of microglia to Aβ. Increased levels of mRNA for TLR2 have been found in the brains of AD patients and AD mouse models [140,141]. Activation of TLR2 in microglia cells by peptidoglycan increases Aβ internalization, inducing the G-protein-coupled receptor FPR2 [141]. Deficiency of TLR2 in a mAPP mouse model led to impaired spatial and nonspatial memory after the third month [142]. *In vitro* experiments using microglia from TLR2 knockdown mice showed a reduction in the expression of TNF-α, iNOS, IL-1β, IL-6, CD11a, CD11b, and CD68 in response to Aβ [143]. TLR2 knockdown mice have a deficiency in the expression of proinflammatory molecules in cortical sections after microinjection of fibrillar Aβ_1–42_ into the cortex [143]. Moreover, colocalization of Aβ_42_ and TLR2 has been shown in primary murine microglia, while the leucine-rich repeat on the N-terminal ectodomain has been identified as the ligand receptor interaction site [144].

Liu *et al.* have also shown that TLR2-deficient bone marrow in chimeric APP transgenic mice treated with Aβ underwent a reduction in the inflammatory response and an increase in Aβ internalization by phagocytosis [144]. Thus, TLR2 inhibition could slow AD pathogenesis by reducing inflammation and enhancing Aβ clearance. However, TLR2 inhibition might interfere with the inflammatory response to other pathogens recognized by this receptor, making it an unlikely therapeutic target.

Increased expression of CD14, TLR2, and TLR4 in AD human brains and animal models has highlighted their role in AD pathology [145]. Treating human monocytes and murine microglia with neutralizing antibodies for CD14, TLR2, and TLR4 followed by fAβ stimulation reduces fAβ binding to cells and the phagocytic response [145]. Microglia cultures from CD14^-/-^, TLR4^-/-^, or TLR2^-/-^ mice treated with fAβ showed a reduction in ROS production, which links these receptors to the oxidative response induced by Aβ. These deficient cells did not activate p38 MAPK in response to fAβ, implicating this pathway in fAβ signaling and microglia activation through TLRs [145]. Further evidence has been provided by studies that showed a reduction in IL-1β and TNF-α production induced by fAβ in microglia after p38 inhibition [146].

TLR9 is another member of the TLR family that is highly expressed when microglia are stimulated with Aβ. Activation of N9 microglia with the TLR9 ligand unmethylated cytosine-guanosine (CpG) increases Aβ uptake through a mechanism that involves the upregulation of FPR2 [147]. A study using a microglia-neuron co-culture system showed that pre-treating microglia with CpG attenuated the neurotoxicity caused by Aβ oligomers [148]. Intracerebroventricular administration of CpG and Aβ oligomers in a transgenic AD model resulted in improvements in cognitive impairment [148]. These results suggest a beneficial role of TLR9 expression in AD pathogenesis.

Overall, research on TLRs suggests that these receptors play a dual role in AD pathogenesis. TLRs are neuroprotective, owing to their contribution to Aβ clearance. Conversely, TLR-triggered inflammatory responses by Aβ can lead to neurotoxic effects. TLRs 2, 4 and 9 have been suggested as therapeutic targets for AD treatment [139,144,148]. However, considering the role of TLRs in the innate immune response to microbial infections and danger signals, modulation of TLR signaling as a potential therapeutic approach presents significant challenges.

## NOD-, LRR- and pyrin domain-containing 3 (NLRP3) inflammasome

Inflammasomes are intracellular multiprotein complexes that sense exogenous and endogenous molecules and are involved in the first line of defense. NLRP3 belongs to the family of the nucleotide-binding domain leucine-rich repeat (LRR)-containing receptors (NOD-like receptors, NLRs) and is a core component of one of the inflammasome complexes. NLRP3 is activated for a variety of molecules including bacterial RNA, toxins, viruses, ATP, uric acid, Aβ, asbestos, silica, and alum [149-151]. This complex is composed of an NLR protein (NLRP3), the adaptor molecule apoptotic speck-containing protein with a card (ASC), and pro-caspase-1. Inflammasomes are the platforms for caspase-1 activation, which mediates the cleavage of inactive IL-1β and IL-18 precursors, an essential step in the secretion of mature cytokine [152].

Activation of microglia cells by Aβ induces the release of the cytokine IL-1β [153]. The first evidence for the role of NLRP3 in IL-1β secretion in AD was provided by Halle *et al.*, who showed that NLRP3 dependent caspase-1 activation occurred in microglia cells after stimulation with Aβ [149]. These authors demonstrated that bone-marrow macrophages from NLRP3-deficient mice failed to release IL-1β in response to Aβ stimuli, and inhibition of Aβ phagocytosis diminished NLRP3-mediated IL-1β release *in vitro*[149]. These results indicate that Aβ phagocytosis is necessary for NLRP3 inflammasome induction of IL-1β. Phagocytosis of Aβ induces lysosomal destabilization and dysfunction, with a consequent cytosolic release of lysosomal enzymes, such as cathepsin B [149]. Cathepsin B seems to be involved in NLRP3 dependent caspase-1 activation, IL-1β secretion and the subsequent release of several proinflammatory and chemotactic mediators [149].

There is an increase in caspase-1 processing in AD individuals, corroborating the role of inflammasome activation in AD [154]. The role of NLRP3 in AD has also been confirmed in AD animal models. APP/PS1/NLRP3^-/-^ and APP/PS1/Casp1^-/-^ mice showed reductions in Aβ deposition and in spatial memory impairment compared with APP/PS1 animals [154].

**Figure 2:**
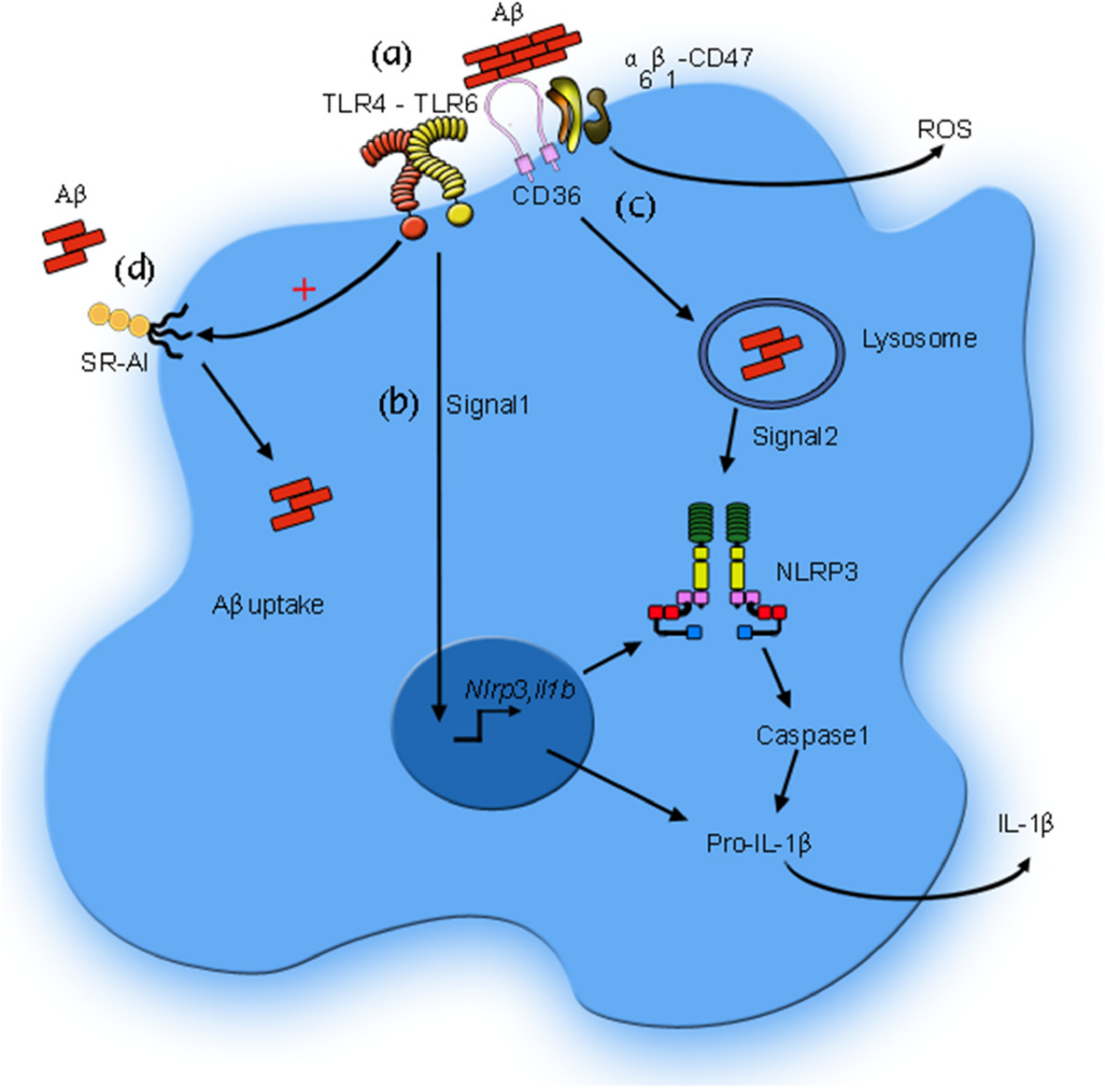
**Cooperation among microglia receptors in Aβ recognition, uptake and signaling. (a)** Aβ fibrils are recognized by the complex CD36-α_6_β_1_-CD47, generating ROS production. The interaction between CD36 and Aβ provides signals for the assembly of the heterodimer TLR4-TLR6 complex. **(b)** CD36-TLR4-TLR6 complex activation constitutes the first signal for the transcription of *Nlrp3* and *il1b*. **(c)** CD36 mediates the internalization of Aβ into the lysosomal compartment. Lysosomal disruption constitutes the second signal for the NLRP3 assembly and the subsequent cleavage of pro-IL-1β, rendering the mature IL-1β. **(d)** The activation of TLR4 also induces the overexpression of SR-AI, which contributes to the clearance of Aβ.

The NLRP3 inflammasome has also been related to the CD36 receptor. CD36 has been shown to play a role in inflammasome activation in AD, atherosclerosis and type 2 diabetes [106,155]. Recognition of oxidized LDL, Aβ, and amylin peptides by CD36 triggers TLR4-TLR6 heterodimer assembly, creating the first signal for NLRP3 activation. CD36 also mediates the internalization of these ligands into the lysosomal compartment, sending a second signal for NLRP3 activation [106,155]. These results further illustrate the cooperation between immune receptors in the response to Aβ in AD (Figure 2).

## Other receptors

Other receptors are involved in AD pathogenesis, such as CD33 and the triggering receptor expressed by myeloid cells 2 (TREM2). Recently, a genome-wide analysis identified different AD risk alleles, including a gene encoding the human protein CD33 [156]. CD33 is a transmembrane protein, a member of the sialic acid-binding immunoglobulin-like lectins, and is expressed in myeloid progenitor cells, including in microglia cells [157-159]. A recent analysis of post-mortem brain samples of patients with AD showed high expression levels of CD33 in microglia surrounding Aβ plaques [160]. *In vitro* assays revealed a negative relationship between CD33 levels and Aβ clearance [160]. Specifically, CD33^-/-^ microglia showed an enhanced capacity to internalize Aβ, whereas the overexpression of CD33 impaired Aβ uptake. AD mice deficient in CD33 exhibited a reduction in Aβ plaques, suggesting that CD33 favors Aβ accumulation [160].

TREM2 is a transmembrane protein that forms a complex with the TYRO protein tyrosine-kinase-binding protein, also known as Dap12. TREM2 is expressed in microglia and neurons and appears to be involved in promoting phagocytosis and in inhibiting the production of inflammatory mediators by these cells [161-163]. TREM2 and its adaptor protein Dap12 are highly expressed in amyloid plaque-associated microglia in APP23 transgenic mice [164]. The role of TREM2 in AD has also been demonstrated in an exome sequencing and whole genome sequencing study [165,166]. A rare mutation in exon 2 of *TREM2*, which encodes for a substitution of histidine for arginine at position 47, represents a risk factor for late-onset AD [165,166]. The loss of function of TREM2 due to this mutation is thought to be the main source of the pathogenic effect of the risk variant [165]. Clinical evidence has shown that carriers of this variant performed worse in cognitive tests than noncarriers and were more susceptible to the development of late-onset CNS diseases [165]. To date, there is an incomplete understanding of specific TREM2 ligands and functions, which makes it difficult to determine the contribution of TREM2 variants to AD progress.

## Conclusions

Existing drugs for AD only treat the symptoms of the disease but do not decelerate or cure AD. Furthermore, the last drug to be approved by the Food and Drug Administration for therapeutic AD treatment was memantine, in 2003. In the last decade, several candidate drugs have failed to reach statistical significance in their primary outcomes. The drugs currently under test in clinical trials are cholinesterase inhibitors, N-methyl-D-aspartate antagonists, inhibitors of Aβ aggregation, and Aβ immunotherapies.

In recent years, the role of microglia in AD pathology has received more attention. In AD, microglia are activated by Aβ, generating a proinflammatory response sustained over time that can cause neuronal death. The damaged neurons release signals that can overactivate microglia, inducing a cycle of neuron damage; this process is known as reactive microgliosis. The Aβ-induced microglia activation pathways are not well understood but the involvement of several receptors in this process is evident. The data discussed here suggest that microglia receptors play a redundant role in the activation of microglia by Aβ. It is unlikely that a single pathway is involved; rather, multiple pathways likely contribute to AD pathogenesis. Table 1 summarizes the receptors discussed here and their potential effects in AD pathogenesis.

**Table 1:** Summary of microglia receptors and their effects in AD pathogenesis

**Receptors**	**Expression in brain cells**	**Role in AD pathogenesis**	**References**
Complement receptors	Neurons, microglia, astrocytes and oligodendrocytes	Microglia activation, cytokine expression and Aβ clearance	[25,26,28,30,31,33,34,37]
Fc receptors	Neurons, microglia, astrocytes and oligodendrocytes	Inflammatory response and Aβ clearance	[41,45]
FPRL1/FPR2	Microglia, astrocytes	Microglia activation, inflammatory response and Aβ internalization	[61,65,66,68,71,167]
SR-A	Microglia, astrocytes	Aβ internalization and clearance	[85,88]
CD36	Neurons, microglia, astrocytes	Microglia recruitment, activation and Aβ phagocytosis	[77,102,106,155]
RAGE	Neurons, microglia and astrocytes	Microglia activation and cytokine expression	[113,115-118]
TLR	Microglia, astrocytes	Microglia activation, inflammatory response and Aβ clearance	[131,132,139,143-145,148]
NLRP3	Microglia	Cytokine expression	[149,153]
CD33	Microglia	Impairs microglia Aβ clearance	[159,160]
TREM2	Microglia, neurons	Aβ clearance	[162-166]

Aβ, amyloid β; AD, Alzheimer’s disease; FPR, formyl peptide receptors; NLRP, nod-like-receptor protein; RAGE, receptor for advanced glycosylation endproducts; SR-A, scavenger receptor A; TLR, toll-like receptor; Trem2, triggering receptor expressed by myeloid cells 2.

Over the last decade, advances have been made in understanding the signal transduction pathways involved in the expression of proinflammatory molecules in AD. Phosphorylation and activation of specific intracellular kinases represent common events in the signaling cascades triggered in Aβ responses. Therefore, those signaling molecules can also be considered targets for new AD drugs. The therapeutic targeting of microglia receptors implicated in the response to Aβ and their associated signaling pathways could reduce the inflammation found in AD. Further studies are necessary to better understand all the molecular mechanisms occurring in this response, so as to establish new therapeutic strategies. The available data strongly suggest that modulating microglia activation and neuroinflammation through microglia receptors could attenuate the Aβ-induced neurodegeneration found in AD patients. However, the immune status and the stage of disease progression are critical factors to consider. The data reviewed here support a multi-targeted immunomodulation approach as a potential treatment to mitigate AD progression and symptoms.

## Abbreviations

Aβ: amyloid β; acLDL: acetylated low-density lipoproteins; AD: Alzheimer’s disease; AGE: advanced glycosylation endproducts; APP: amyloid precursor protein; CNS: central nervous system; CpG: cytosine-guanosine; CSF: cerebrospinal fluid; ERK: extracellular signal-regulated kinases; fAβ: fibrillar amyloid β; FcR: Fc receptor; FPR: formal peptide receptor; Ig: immunoglobulin; LDL: low-density lipoprotein; LPS: lipopolysaccharide; LRP: LDL receptor-related protein; LRR: leucine-rich repeat; MAC: membrane attack complex; MAPK: mitogen-activated protein kinase; MPL: monophosphoryl lipid A; NLR: NOD-like receptor; NLRP3: NOD-, LRR- and pyrin domain-containing 3; RAGE: receptor for advanced glycosylation endproducts; ROS: reactive oxygen species; SR: scavenger receptor; TLR: toll-like receptor; TNF-α: tumor necrosis factor-α; Trem2: triggering receptor expressed by myeloid cells 2.

## Competing interests

The authors do not have any competing interests.

## Authors’ contributions

Both authors prepared the manuscript and figures and approved the final manuscript.
